# ‘Timed Up and Go’ test: Age, gender and cognitive impairment stratified normative values of older adults

**DOI:** 10.1371/journal.pone.0185641

**Published:** 2017-10-03

**Authors:** Azianah Ibrahim, Devinder Kaur Ajit Singh, Suzana Shahar

**Affiliations:** 1 Physiotherapy Programme, School of Rehabilitation Sciences, Faculty of Health Sciences, Universiti Kebangsaan Malaysia, Jalan Raja Muda Abdul Aziz, Kuala Lumpur, Malaysia; 2 Pantai Integrated Rehab Services Sdn Bhd, Pandan Indah, Kuala Lumpur, Malaysia; 3 Community Rehabilitation and Aging Research Centre, Faculty of Health Sciences, Universiti Kebangsaan Malaysia, Jalan Raja Muda Abdul Aziz, Kuala Lumpur, Malaysia; Nathan S Kline Institute, UNITED STATES

## Abstract

**Aims:**

The aim of this study was to establish ‘Timed up and Go’ test (TUG) normative data among community dwelling older adults stratified based on cognitive status, gender and age groups.

**Methods:**

A total of 2084 community dwelling older adults from wave I and II were recruited through a multistage random sampling method. TUG was performed using the standard protocol and scores were then stratified based on with and without mild cognitive impairment (MCI), gender and in a 5-year age groups ranging from ages of 60’s to 80’s.

**Results:**

529(16%) participants were identified to have MCI. Past history of falls and medical history of hypertension, heart disease, joint pain, hearing and vision problem, and urinary incontinence were found to have influenced TUG performance. Cognitive status as a mediator, predicted TUG performance even when both gender and age were controlled for (*B* 0.24, 95% CI (0.02–0.47), *β* 0.03, *t* 2.10, *p* = 0.36). Further descriptive analysis showed, participants with MCI, women and older in age took a longer time to complete TUG, as compared to men with MCI across all age groups with exceptions for some age groups.

**Conclusion:**

These results suggested that MCI needs to be taken into consideration when testing older adults using TUG, besides age and gender factors. Data using fast speed TUG may be required among older adults with and without MCI for further understanding.

## Introduction

The number of older adults in the world is estimated to double from year 2000 and 2050 to 22% [1]. Majority of these older adults (62.3%) are projected to be living in Asia by the year 2050 [2]. Similar trends are seen in Malaysia and it will be categorised as an ageing nation by the year 2035 as this group of population is expected to occupy 15% of the total population [3]. In 2050, the eighty-and-older population is projected to be quadrupled [2].

Disabilities among older adults are mainly precipitated by cognitive impairments [4]. Mild cognitive impairment (MCI) has been identified as a transition phase between normal cognitive ageing and early dementia [5]. Approximately, 15 to 30% older adults globally and in 22% in Malaysia are reported to have MCI [6,7].

Limited functional mobility is a major concern in older adults with MCI. Changes in gait among older adults are due to reduction in muscle mass and muscle strength, deterioration of postural stability and vestibular function [8,9]. In older adults with MCI, motor control as in gait can be further affected by impairments of primary motor cortex [10]. Evidence regarding association between gait and MCI indicated that older adults with MCI had poorer performance in their gait assessment which was measured using gait parameters such as velocity [11,12], stride length [13] and coefficient of variation [14,15]. These gait parameters are sensitive and accurate measures to detect alterations in gait among older adults with MCI. However, this test is not viable for large scale community screenings due to its cost, duration and training that may prove to be costly.

Simple physical performance assessment tools such as TUG [12,16–19], gait speed [12,18,20] and 4-minute walk [20] tests have been used among older adults with MCI. TUG test was reported to be most consistent in differentiating older adults with and without MCI. Older adults with MCI were noted to have poorer TUG performance [12,16–18] and cognitive impairment was identified as an independent determinant of TUG score [21]. This could be explained by the tasks in TUG test which places additional cognitive challenges such as straight walking on executive functioning via initiation and sequencing; transfer and turning on cognitive processing speed [22,23]. Multiple cognitive domains that includes attention, memory, visual spatial ability and executive functions are further challenged when performing a walking task to maintain balance and prevent falls [24]. Although TUG test is simple, its performance requires integration of many systems and can be considered as complex [23], more so in older adults with cognitive impairment.

Use of local normative data of the specific population is recommended for more meaningful interpretation of TUG results [23]. Physiological and anthropometric measures, such as height and limb length vary across ethnicity and it is associated with physical performance. TUG normative data is available among Spanish community dwelling older adults with an inclusion of those with cognitive impairment assessed using global deterioration test [21]. However, it is not certain whether TUG performance among older adults with and without MCI differs significantly. Therefore, the aim of this study was to identify the normative data of TUG test among community dwelling older adults with and without MCI, according to cognitive status, gender and age group. Besides cognitive status, TUG will be stratified based on gender and age, as recommended by Steffen, Hacker & Mollinger (2002) [25].

## Methods

This cohort prospective longitudinal study involved four states in Malaysia, which were chosen based on density of older adult population [26]. The study was carried out in two phases. The first wave was held from February 2012 to February 2013 and second wave was carried out from November 2014 to August 2015. The selected states were Johor (southern zone), Perak (northern zone), Selangor (central zone) and Kelantan (east coast zone). Multistage random sampling performed by Statistics Department of Malaysia was utilised, as this study is part of a large scale longitudinal study on neuroprotective model for healthy longevity. Sampling frame and design are as explained in our teams’ recent study [27]. Race proportion in this study is similar to the Malaysian population and therefore, could be representative of Malaysians [28]. Ethical approval for this study was obtained from Medical Research and Ethics Committee of Universiti Kebangsaan Malaysia (UKM 1.5.3.5/244/NN-060-2013).

The inclusion criteria included community dwelling older adults aged between 60–90 years old, able to walk 7metres and get in or out of a chair with or without assistive device. Participants were excluded if they were unable to comprehend and follow instructions (Malay, English, Mandarin, or Tamil language), having any acute illnesses, having pain in any segment greater than 2 on a 10-point verbal analogue scale, had recent fractures of vertebral or lower limbs or recent lower extremity surgery (in the past 6 months). Participants with known neurological or musculoskeletal diagnosis that could account for possible imbalance and falls (such as cerebrovascular accident, Parkinson disease, or lower-extremity joint replacements), diagnosed with psychological or psychiatric disorders and with severe cognitive impairments (Mini Mental State Examination score less than 15 [29]) were also excluded from this study.

All participants were provided with informed written information regarding the study and written consent was obtained. A structured interview was administered to obtain the socio-demographic data which included age, gender, race and self-reported medical conditions (hypertension, diabetes, heart disease, cataract/glaucoma, joint pain, gout, hearing and vision problem, and urinary incontinence). History of falls was obtained based on previous falls in the past 18 months. Participants’ anthropometric measurements of body weight and height were taken.

MCI was identified according to studies by Peterson [30] and Shahar et al. [27] which comprised of subjective cognitive impairment- identified by the question ‘Do you have memory complaints?”, objective cognitive impairment- scored at more than 1 from below the norm mean for either Rey Auditory Verbal Learning Test (RAVLT) or Digit Span Test, no dementia- confirmed by doctor, no limitations in basic activities of daily living, no or minimal functional limitations- indicated using Lawton Instrumental Activities of Daily Living Scale and intact global cognition- scored ≥19/30 for Malay version of Mini Mental Examination State (MMSE).

Participants were required to perform TUG, instructed to rise from an armless chair (46cm height), walk 3 metres and turned around at a cone placement, walk back, and sit again [31]. They were instructed to walk at a normal pace with or without walking aids and shoes. Time was recorded when participants’ buttocks were lifted off the chair to stand and ceased when the buttocks touched the seat when returning to sitting position. The test was performed twice consecutively, and the averages of the scores in seconds were used for further analysis. This test was carried out by a physiotherapist and a trained research assistant. TUG has excellent intra-rater reliability in community-dwelling older adults (intra-class coefficient of 0.94) [32] and moderate to excellent validity in older adults with and without MCI (Pearson correlation 0.64–0.74) [33].

### Statistical analysis

Longitudinal TUG data from wave 1 and wave 2 were merged in order to produce normative data, as done previously in developing growth chart standard [34]. Participants were categorized in 5-year age groups, ranging from group of 60–64 to 80–84. Mean TUG comparison against cognitive status, gender and age group variation was analyzed by means of independent sample t-test or one-way analysis of variance (ANOVA) and multifactorial ANOVA. Factors which were found to be significantly different in TUG performance on t-test and ANOVA, besides cognitive status, age and gender, were included as covariates in the following multifactorial ANOVA test [35]. Further mediator analysis was carried out to test whether cognitive status acted as a mediator in TUG performance. Finally, mean, standard error, and 95% confidence interval of the TUG scores were then reported, to establish the normative values of TUG. Analysis was carried out based on cognitive status and further stratified according to gender and age group.

## Results

Participants’ sociodemographic data is as depicted in Tables 1 and 2. In wave 1, 1005 men and 1079 women, with mean age of 69.2(±5.9) years and 68.2(±6.0) years of age, participated in this study. Upon MCI classification, 16% of the participants were classified as MCI. Most of the participants with past history of falls were women (22%), which contributed to 18% of total participants with history of falls.

**Table 1:** Socio demographic data—Wave I [measured as mean ± SD or n (%)].

Variables	Men (n = 1005)	Women (n = 1079)	Total (n = 2084)
**Age** (mean ± SD)	69.2±5.9	68.2±6.0	68.7±6.0
**Age group** n(%)			
60–64 years	256 (25.5)	369 (34.2)	625 (30.0)
65–69 years	294 (29.3)	320 (29.7)	614 (29.5)
70–74 years	253(25.2)	216 (20.0)	469 (22.5)
75–79 years	150(14.9)	129 (12.0)	279 (13.4)
≥80 years	52 (5.2)	45 (4.2)	97 (4.7)
**Nationality** n(%)			
Malay	660 (65.7)	629 (58.3)	1289 (61.9)
Indian	49 (4.9)	54 (5.0)	103 (4.9)
Chinese	293 (29.2)	395 (36.6)	688 (33.0)
Others	3 (0.3)	1 (0.1)	4 (0.2)
**Weight** (mean ± SD)	64.5±11.7	57.6±11.4	60.9±12.0
**Height** (mean ± SD)	162.1±6.5	150.4±5.9	156.0±8.5
**Cognitive status** n(%)			
MCI	178 (17.7)	134 (12.4)	312 (15.0)
Non-MCI	827 (82.3)	945 (87.6)	1772(85.0)
**Past History of Falls** n(%)			
Had history of fall	150 (14.9)	232 (21.5)	382 (18.3)
No history of fall	855 (85.1)	847 (78.5)	1702 (81.7)
**Having Medical Illness** n(%)			
Hypertension	454 (45.2)	564 (52.3)	1018 (48.9)
Diabetes	269 (26.8)	274 (25.4)	543 (26.1)
Heart Disease	128 (12.7)	80 (7.4)	208 (10.0)
Cataract/Glaucoma	88 (8.8)	100 (9.3)	188 (9.0)
Joint pain	215 (21.4)	283 (26.2)	498 (23.9)
Gout	64 (6.4)	22 (2.0)	86 (4.1)
Hearing and vision problem	138 (13.7)	100 (9.3)	238 (11.4)
Urinary Incontinence	136 (13.5)	62 (5.8)	198 (9.5)
**TUG** (mean ± SD)(s)	10.8±2.5	11.5±2.7	11.1±2.6

TUG = timed up and go; MCI = mild cognitive impairment, SD = standard deviation, s = second

**Table 2:** Socio demographic data—Wave 2 [measured as mean ± SD or n (%)].

Variables	Men (n = 661)	Women (n = 640)	Total (n = 1301)
**Age** (mean ± SD)	70.6±5.7	69.1±5.5	69.9±5.7
**Age group** n(%)			
60–64 years	108 (16.3)	146 (22.8)	254 (19.5)
65–69 years	207 (29.3)	226 (35.3)	433 (33.3)
70–74 years	181 (25.2)	161 (25.2)	342 (26.3)
75–79 years	121 (14.9)	77 (12.0)	198 (15.2)
≥80 years	44 (5.2)	30 (4.7)	74 (5.7)
**Nationality** n(%)			
Malay	445 (67.3)	371 (58.0)	816 (62.7)
Indian	35 (5.3)	25 (3.9)	60 (4.6)
Chinese	181 (27.4)	244 (38.1)	425 (32.7)
**Weight** (mean ± SD)	64.4±12.3	58.0±11.4	61.3±12.3
**Height** (mean ± SD)	161.7±6.5	150.5±5.9	156.3±8.4
**Cognitive status** n(%)			
MCI	130 (19.7)	87 (13.6)	217 (16.7)
Non-MCI	531 (80.3)	553 (86.4)	1084 (83.3)
**Past History of Falls** n(%)			
Had history of fall	92 (13.9)	147 (23.0)	239 (18.4)
No history of fall	569 (86.1)	492 (76.9)	1061 (81.6)
**Medical History** n(%)			
Hypertension	314 (47.5)	334 (52.2)	648 (49.8)
Diabetes	177 (26.8)	164 (25.6)	341 (26.2)
Heart Disease	76 (11.5)	31 (4.8)	107 (8.2)
Cataract/Glaucoma	70 (10.6)	71 (11.1)	141 (10.8)
Joint pain	151 (22.8)	168 (26.3)	319 (24.5)
Gout	40 (6.1)	19 (3.0)	59 (4.5)
Hearing and vision problem	35 (30.0)	30 (4.7)	65 (5.0)
Urinary Incontinence	99 (15.0)	29 (4.5)	128 (9.8)
**TUG** (mean ± SD)(s)	11.2±2.4	11.9±2.6	11.6±2.5

TUG = timed up and go; MCI = mild cognitive impairment, SD = standard deviation, s = second

661 men and 641 women from wave I participated in wave 2 of this study. Older adults classified as MCI increased by 1.7%, to 16.7%. A total of 18.4% of the participants in wave II had a history falls in the past 18 months. Self-reported medical history showed that percentage of conditions in older adults had increased from wave I to wave II, except for heart disease, hearing and vision problems.

As shown in Table 3, participants with MCI significantly took longer time (11.6±2.5s) as compared to those without MCI (11.3±2.6s) (p<0.05). Similarly, women (11.6±2.7s) were slower than men (11.0±2.5s) (p<0.001) in performing TUG. Time taken to perform TUG also increased significantly (p<0.001) with age. In addition, older adults having history of falls, self-reported medical condition of hypertension, heart disease, joint pain, vision/hearing impairment, incontinence and cataract/glaucoma had significantly poorer TUG performance compared to those without having any medical conditions. These factors had an influence on TUG performance and therefore were taken as covariates in the following analysis.

**Table 3:** Mean differences in pooled data of TUG score performance among older adults based on cognitive, gender and age.

Variables	TUG score (s)				Difference in TUG Performance (p-value)
	n	%	Mean	SD	
**Cognitive**					
Without MCI	2856	84.4	11.3	2.6	**0.007** ^a^
MCI	529	15.6	11.6	2.5	
**Gender**					
Men	1666	47.7	11.0	2.5	**<0.001** ^a^
Women	1719	52.3	11.6	2.7	
**Age group** (years)					
60–64	879	23.9	10.4	2.3	**<0.001** ^b^
65–69	1047	30.2	11.0	2.4	
70–74	811	24.1	11.4	2.6	
75–79	477	15.7	12.6	2.7	
>80	171	6.1	13.6	2.5	
**Past History of Falls n(%)**					
Had history of fall	621	18.3	11.6	2.6	**0.001** ^a^
No history of fall	2764	81.7	11.2	2.7	
**Medical History**					
Hypertension*					**<0.001** ^a^
Yes	303	9.8	11.85	2.65	
No	3082	90.2	11.25	2.59	
Diabetes					0.25 ^a^
Yes	884	26.1	11.39	2.59	
No	2501	73.9	11.27	2.60	
Heart Disease					**0.02** ^a^
Yes	315	9.3	11.62	2.64	
No	3070	90.7	11.27	2.59	
Cataract/Glaucoma					0.43 ^a^
Yes	329	9.7	11.42	2.83	
No	3056	90.3	11.29	2.57	
Joint pain					**<0.001** ^a^
Yes	817	24.1	11.78	2.61	
No	2568	75.9	11.15	2.57	
Gout					0.07 ^a^
Yes	145	4.3	11.69	2.77	
No	3240	95.7	11.28	2.59	
Hearing and vision problem					**<0.001** ^a^
Yes	303	9.0	11.85	2.65	
No	3082	91.0	11.25	2.59	
Urinary Incontinence					**0.003** ^a^
Yes	326	9.6	11.71	2.57	
No	3059	90.4	11.26	2.60	

TUG = timed up and go; s = seconds; MCI = mild cognitive impairment; SD = standard deviation;

^a^Independent t-test;

^b^ANOVA,

Table 4 depicts multifactorial interaction in TUG performance, adjusted for hypertension, heart disease, joint pain, vision/hearing impairment, incontinence and history of falls. There was no interaction effect of age and gender. However, significant main effect for age and gender (p<0.05) in TUG performance was demonstrated. This indicates that time taken for TUG performance was significantly longer in women compared to men and in oldest age group compared to younger age group. Post-hoc test showed that older adults in younger age were significantly faster than the older age groups, consistently. Generally, an increasing trend of time taken for TUG performance across age groups and were similar in both genders.

**Table 4:** Multifactorial interaction in TUG score performance.

Variables	df	F	Sig
Gender	1	22.5	<.0001
Age	4	42.2	<.0001
Cognitive Status	1	0.2	.689
Age*Gender	4	1.0	.426
Cognitive Status*Gender	1	4.0	.046
Cognitive Status*Age	4	2.7	.027
Cognitive Status*Gender* Age	4	0.5	.714

Adjusted for Hypertension, Heart Disease, Joint Pain, Vision/Hearing Impairment, Incontinence, History of fall

Conversely, cognitive status significantly interacted with age (F _[4,3357]_ = 2.7, *p* = 0.027) and gender (F _[1,3357]_ = 4.0, *p* = 0.046) on TUG performance. However, cognitive status independently had no significant effect on TUG performance. This indicates that among older adults who were older in age and with MCI significantly took longer time compared to those without MCI in TUG performance. Gender wise, women with MCI took significantly longer time to perform TUG compared to women without MCI. Nonetheless, three-way interaction of gender, age and cognitive status was not significant.

Further analysis for mediation using regression analysis was performed to test if cognitive status mediated the effect of gender and age on TUG performance. The first regression showed that gender (*B* 0.88,95%CI (0.72–1.05), *β 0*.*17*, *t* 10.55, *p*<0.001) and age (*B 0*.*77*,95%CI (0.71–0.84), *β* 0.34, *t* 21.33, *p*<0.001) had an effect on TUG. The second regression performed demonstrated that gender and age predicted the mediator (cognitive status). Both age and gender were respectively found to be positively (*B* 0.02,95%CI (0.01–0.03), *β* 0.07, *t* 3.81, *p*<0.001) and negatively (*B*-0.05,95%CI (-0.08- -0.03), *β* -0.07, *t* -4.10, *p*<0.001) associated with cognitive status.

Table 5 depicts hierarchical regression analysis using TUG performance as dependent variable, gender and age as predictors in first step and cognitive in the second step. Results showed that in the first step, both gender (*B* 0.88,95%CI (0.72–1.05), *β*0.17, *t* 10.55, *p*< 0.001) and age (*B*0.77,95%CI (0.70–0.84), *β* 0.77, *t* 21.33, *p*< 0.001) predicted TUG performance. When cognitive status was controlled as a mediator, predictability was slightly reduced but was still significant for gender (*B* 0.89,95%CI (0.73–1.06), *β* 0.17, *t* 10.68, *p*<0.001) and age (*B* 0.76,95%CI (0.69–0.83), *β* 0.34, *t* 21.16, *p*<0.001). Cognitive status, as a mediator, predicted TUG performance even when both gender and age were controlled for (*B* 0.24,95%CI (0.02–0.47), *β* 0.03, *t* 2.10, *p* = 0.36). Cognitive status improved the prediction of TUG performance in addition the independent variables (gender and age) (Δ*R*^*2*^ 0.001, *F* 4.40, *p* = 0.36).

**Table 5:** Predictors for TUG using cognitive status as mediator.

Predictor	Step1	Step2	
	*B*	*β*	95%CI
**Age**	0.77***	0.76***	0.69–0.83
**Gender**	0.88***	0.89***	0.73–1.06
**Cognitive status**		0.24*	0.15–0.47
***R***^***2***^	13.4	13.4	
***F***	111.4	4.4	
**Δ*R***^***2***^		0.001	
**Δ*F***		4.4*	

*p<0.5,

**p<.01

***<.001

**Fig 1:**
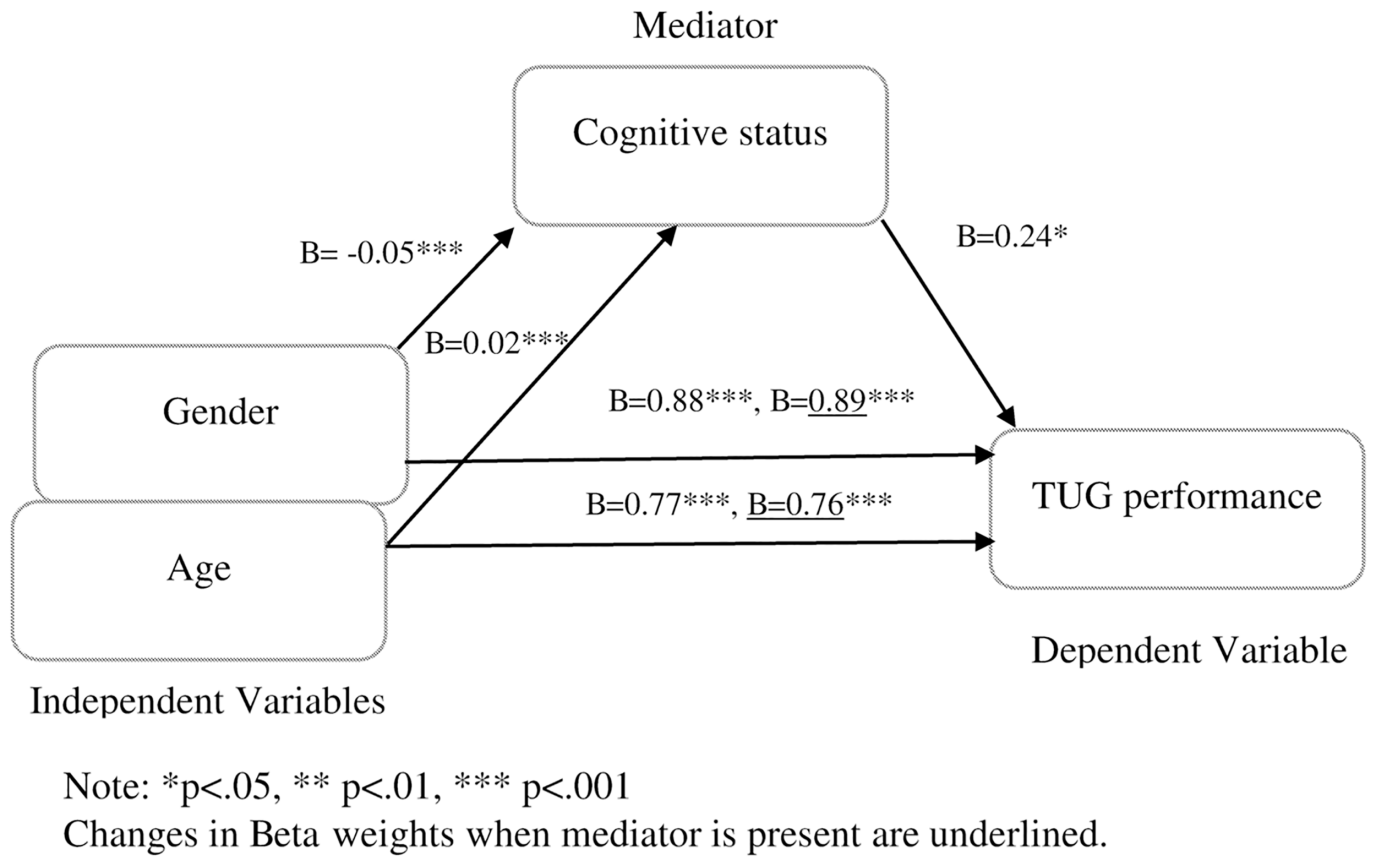
Mediation model showing the effects of gender and age on TUG performance with cognitive status as the mediator.

Sobel test showed that the complete pathway gender (independent variable) to cognitive(mediator) to TUG performance (dependent variable) was significant (*z* -2.66, *p* <0.01). The complete pathway from age (independent variable) to cognitive status (mediator) to TUG performance (dependent variable) was also significant (*z* 2.22, *p* 0.03). Therefore, indicating that cognitive status partially mediates the effect of gender and age on TUG performance. This result suggest that gender and age contribute directly to explain variation in TUG performance and indirectly via cognitive status (Fig 1). Therefore, cognitive status was included as a factor in establishing TUG normative data.

Descriptive analysis (Table 6) showed that women in older age groups with MCI took longer time to complete TUG across all age groups. However, some of the age groups with MCI completed TUG at a shorter duration compared to those without MCI i.e., age group 80–84 years for men and 70–74 years, 75–79 years and 80–84 years for women. For instance, men with MCI in age group of 80–84 years had better TUG performance compared to men without MCI in age group of 80–84 years. Some older adults in certain age groups with MCI also appeared to be faster in TUG performance compared to younger age groups. Women with MCI in age group 70–74 were faster in TUG performance compared to some younger age groups (women with MCI in age group 65–69 years). Similarly, men with MCI in age group 65–69 in a shorter time in comparison to men with MCI in age group 60-64years.

**Table 6:** Timed Up and Go scores by cognitive status, age group and gender.

	Without MCI(n = 2,856)										With MCI(n = 529)									
	Men(n = 1,358)					Women(n = 1,498)					Men(n = 308)					Women(n = 221)				
Age Range (years)	60–64	65–69	70–74	75–79	80–84	60–64	65–69	70–74	75–79	80–84	60–64	65–69	70–74	75–79	80–84	60–64	65–69	70–74	75–79	80–84
**N**	314	418	348	205	73	458	475	326	172	67	50	83	86	66	23	57	71	51	34	8
**Mean(s)**	9.9	10.4	10.9	12.2	13.2	10.7	11.4	12.0	13.1	14.3	11.0	10.9	11.2	12.3	12.9	10.9	11.9	11.6	12.8	13.1
**Standard Error**	0.1	0.1	0.1	0.2	0.3	0.1	0.1	0.1	0.2	0.3	0.3	0.3	0.3	0.3	0.5	0.3	0.3	0.3	0.4	0.8
**95% CI**	9.6–10.1	10.2–10.7	10.6–11.1	11.9–12.6	12.6–13.7	10.5–10.9	11.2–11.7	11.7–12.2	12.7–13.4	13.7–14.8	10.4–11.7	10.4–11.4	10.7–11.7	11.7–12.8	11.9–13.8	10.3–11.6	11.3–12.4	10.9–12.3	12.0–13.6	11.4–14.7

s = seconds

## Discussion

The aim of our study was to identify normative data of TUG performance among community dwelling older adults, according to with and without MCI, gender and age groups. The results showed that TUG performance was moderated by MCI x gender and MCI x age. However, age x gender x MCI and MCI alone were not found to moderate TUG performance. To the best of our knowledge, no previous information is available regarding multifactorial interaction of age, gender and MCI on TUG performance more so, in establishing normative data of TUG based on these factors.

Our findings showed that TUG performance was moderated by age or gender but not age x gender. Decline in physical performance with aging such as muscle strength, and men having better physical performance compared to women are known facts [25, 36–37]. Meanwhile, result for age x gender on TUG performance suggested that TUG performance pattern may be similar among older adults across genders. Similar results were found in a 10-year prospective Swedish study among older adults aged 50 to 80 years [38]. The authors linked these results to similar age-related decline rate in physical performance namely, gait, balance and hand grip strength in both genders [38].

MCI solely or in three-way interactions with age and gender did not affect TUG performance. However, if gender or age is considered along with MCI, it appeared to act as TUG performance moderator. This result suggested that MCI had an influence on TUG performance but not on its own. Our findings are supported by a previous study showing that older adults with MCI took longer time to perform TUG, although it was not significant [19]. More sensitive tools may be required to detect micro changes in functional mobility among older adults with MCI as summarised in a recent review [15]. Moreover, age and gender were demonstrated to be associated with the magnitude of cognitive deterioration [39].

Consistent with information on normative values of TUG performance among older adults without MCI [21,40], we found that TUG performance in older adults is dependent on age and gender. Data stratification by gender and age in our study showed that older women consistently took longer time to perform TUG across all age groups, compared to men. This trend whereby, women been slower than men with increase in age groups, is similar to the work of Pondal & Ser [21], studied among 527 Spanish community dwelling older adults. This finding however is contradictory to the results of the study by Steffen, Hacker & Mollinger [25] involving 96 community Canadian dwelling older adults.

TUG scores of the youngest to oldest age group ranged between 8 to 11 seconds for men and 10 to 12 seconds for women [21]. In contrast, TUG scores for women and men were reported to be similar with a score of 8 and 9 seconds for age group 60 to 69 years and 70 to 79 years, respectively [25]. Only in age group 80 to 89 years, a difference in TUG score was noted between women (11 seconds) and men (10 seconds). Small sample size in each subgroups which ranged from 8–22, may have influenced the results [25].

TUG score for men and women in age group of early 70 years to 80 was approximately 9 and 11 seconds respectively [21]. In comparison, our study participants achieved similar TUG performance in the early 60-years age group, indicating slower TUG performance compared to Spanish older adults. The difference is possible due to many factors. Firstly, it may be due to variation in race. It was found that Japanese adults and those residing in Western Europe countries had faster walking speed [41]. While, adults from non-industrialized countries such as Middle East, Latin America and Asia had slower walking speed [41]. Secondly, methodological difference may exist in the different studies. For example, in the Spanish study [21], older adults with osteoarticular disease and with gait disturbances were excluded. However, to allow generalisation of the results in our study, factors such as osteoarticular diseases which is a common condition in older adults was included with consideration of the level of pain [42]. In our study, about 25% of the participants had joint pain. Lastly, TUG instructions differed.

The several strengths in our study, included the large and robust sampling method which is similar to national data [26], allowing generalisation and also robust characterisation of MCI. TUG has been used for decades and a number of reference values has been established. However, most of the values apply for older adults with age of 65 years and above. Thus, this study adds to the TUG normative data of older adults at the age group of 60 to 65 years, which is lacking in most previous studies. The limitation of our study was that the sample size of older adults with MCI was limited and it may not be representative of the group. This could have also resulted in inconsistent trends of TUG performance.

In future studies, assessing TUG performance with fast speed [43] is warranted as this test may further challenge cognitive ability. It is hoped that clinically, a meaningful difference between older adults with and without MCI may be possible, while still maintaining simplicity and practicality of the test for large-scale screening. In addition, it will be beneficial to further examine if TUG performance used to screen for functional mobility will be similarly useful to detect early MCI among older adults as the association between mobility and cognition is bidirectional. Older adults without dementia and with abnormal gait was demonstrated to have higher risk of developing dementia after seven years of follow up. There may be a potential to use mobility test in identifying older adults with MCI as it appears much earlier compared to difficulty in performing activities of daily living [44].”

## Conclusions

This is among the first large scale study designed to determine normative values of TUG in community dwelling older adults based on cognitive status, gender and age groups. This study has found that generally older adults with MCI took longer time to accomplish TUG. Hence, TUG normative values presented in this study might be useful to serve as reference for community dwelling older adults’ performance. Although the interaction (gender x age x cognitive status) was not statistically significant, detailing TUG performance based on cognitive status, gender and age will provide researchers and clinicians with more precise representation of functional mobility among older adults with and without MCI, using a simple test. Besides that, therapists are suggested to screen older adults for MCI prior to assessing TUG test for accurate interpretation. Early identification of mobility impairment especially among older adults with MCI will allow more gain from early intervention management.
